# The Macrophage Response to *Mycobacterium tuberculosis* and Opportunities for Autophagy Inducing Nanomedicines for Tuberculosis Therapy

**DOI:** 10.3389/fcimb.2020.618414

**Published:** 2021-02-08

**Authors:** Retsepile E. Maphasa, Mervin Meyer, Admire Dube

**Affiliations:** ^1^ Infectious Disease Nanomedicine Research Group, School of Pharmacy, University of the Western Cape, Cape Town, South Africa; ^2^ DST/Mintek Nanotechnology Innovation Centre, Biolabels Node, Department of Biotechnology, University of the Western Cape, Cape Town, South Africa

**Keywords:** *Mycobacterium tuberculosis*, innate immunity, autophagy and tuberculosis, host directed therapies, immunotherapeutic nanoparticles, xenophagy, LC3-associated phagocytosis, apoptosis and tuberculosis

## Abstract

The major causative agent of tuberculosis (TB), i.e., *Mycobacterium tuberculosis (Mtb)*, has developed mechanisms to evade host defense responses and persist within host cells for prolonged periods of time. *Mtb* is also increasingly resistant to existing anti-TB drugs. There is therefore an urgent need to develop new therapeutics for TB and host directed therapies (HDTs) hold potential as effective therapeutics for TB. There is growing interest in the induction of autophagy in *Mtb* host cells using autophagy inducing compounds (AICs). Nanoparticles (NPs) can enhance the effect of AICs, thus improving stability, enabling cell targeting and providing opportunities for multimodal therapy. In this review, we focus on the macrophage responses to *Mtb* infection, in particular, the mechanistic aspects of autophagy and the evasion of autophagy by intracellular *Mtb*. Due to the overlap between the onset of autophagy and apoptosis; we also focus on the relationship between apoptosis and autophagy. We will also review known AICs in the context of *Mtb* infection. Finally, we discuss the applications of NPs in inducing autophagy with the intention of sharing insights to encourage further research and development of nanomedicine HDTs for TB therapy.

## Introduction

TB caused by an infection from the bacillus *Mycobacterium tuberculosis (Mtb)* is among the top ten leading causes of death globally, and is the main cause of death from a single infectious agent. An estimated 1.7 billion people across the world are infected with *Mtb* and in 2018, approximately 10 million new cases were reported, corresponding to 130 cases per 100,000 population (World Health Organization, 2019). Geographically, the majority of TB cases are found mostly in South-East Asia, Africa, and the Western Pacific. The eight countries; India, China, Indonesia, the Philippines, Pakistan, Nigeria, Bangladesh and South Africa account for two thirds of all cases globally (World Health Organization, 2019).

TB commonly affects the lungs, where it is called pulmonary TB (PTB) but can also affect other organs throughout the body and in this case; it is known as extra-pulmonary TB (EPTB) (World Health Organization, 2018). It is estimated that between 5 and 15% of the estimated 1.7 billion people (about 20% of the world population) is infected with *Mtb* will develop TB during their lifetime (World Health Organization, 2018). However, the probability of developing TB is greater for people living with HIV and also higher for people who smoke, consume alcohol, have diabetes, and/or are under-nourished (World Health Organization, 2019). The current drug therapy for TB is a six-month regimen that consists of a two month intensive phase, where patients receive four first-line antibiotics [i.e., ethambutol hydrochloride (EMB), isoniazid (INH), rifampicin (RIF), and pyrazinamide (PZA)], followed by a four month continuous phase, where patients receive RIF and INH (Grange and Zumla, 2002; Nasiruddin et al., 2017; Bekale et al., 2018).

The lengthy multidrug therapy has been shown to overwhelm patients with high pill load and toxic side effects, resulting in a considerable reduction inpatient compliance and adherence to the medication (Dartois, 2014). This therefore increases the potential risk for the development of drug-resistant strains in noncompliant patients (Kimmey and Stallings, 2016; Kaufmann et al., 2018). As a result, drug-resistant TB remains a major public health hazard. In 2018, approximately 500,000 new cases of RIF-resistant TB (RR-TB) (of which 78% had multidrug-resistant TB) were reported (World Health Organization, 2019).

India, China and the Russian Federation were found to be the three countries with the largest share of the global burden of RR-TB, with each having 27%, 14% and 9%, respectively. In addition, 18% of treated cases in 2018, were either multidrug-resistant TB (MDR-TB) or RIF-resistant TB (RR-TB), with the highest proportions (>50%) of these cases being in countries of the former Soviet Union (World Health Organization, 2019). MDR-TB plus resistance to at least one of the fluoroquinolones and one of the injectable agents used in MDR-TB treatment regimens is known as extensively drug-resistant TB (XDR-TB). In 2018, at least one case of XDR-TB was reported by each of the 131 WHO Member States. However, over the past 15 years, the average proportion of MDR-TB cases with XDR-TB reported by 128 countries was 6.2% of all TB cases (World Health Organization, 2019).

Macrophages are most common host cells of *Mtb* in pulmonary TB. Therefore, the successful treatment of pulmonary TB depends on the efficiency of TB drugs to infiltrate complex lung lesions, penetrate the cell membrane of macrophages and other host cells and finally, for the drugs to be taken up by the intracellular *Mtb* (Dartois, 2014). A study by Gurumurthy et al., demonstrated that TB drugs are malabsorbed, rifampin in particular, was found to be malabsorbed in patients with advanced HIV and diarrhea and cryptosporidial infection. The bioavailability of INH was found to be more reduced in rapid acetylators, while the absorption of EMB and PZA was also reduced in the same patient group. These results suggest that the malabsorption of anti-TB drugs in TB patients with HIV, and diarrhea could be one of the factors affecting the success of anti-TB therapy in this patient cluster (Gurumurthy et al., 2004).

Anti-TB fixed dose combination (FDC) drugs and products have been described as unstable in formulations due to chemical interactions between the drugs, this incompatibility can also lead to a reduction in the bioavailability of the drugs during oral administration. Therefore, in a previous study by Aucamp et al., interactions as well as possible incompatibilities between the four commonly prescribed commercial anti-TB FDC drugs were investigated at a temperature of 50°C. The study revealed that INH and RIF are chemically incompatible (Aucamp et al., 2019). Anti-TB drugs should also be taken on an empty stomach to reduce interaction with various foods, which results in the absortion of these drugs being reduced. In addition, anti-TB drugs also interact with other drugs when taken as part of a combination treatment, this interaction with foods and/or other drugs has been shown to increase the side effect inducing capacity of anti-TB drugs e.g., interaction between isonizid with levodopa, or carbohydrate rich foods (Arbex et al., 2010).

First-line and second-line TB drugs have been shown to posess different intracellular uptake properties, and intracellular to extracellular (I/E) ratios ranging between 0.1 to >20. In both *in vitro* and *in vivo* studies, aminoglycosides and β-lactams (antibiotics that are used to treat XDR TB) were found to have I/E ratios that were below 1, the I/E ratio of INH was found to be approximately 1. On the other hand, the I/E ratio ofRIF ranged between two and five, while the I/E ratios of ETH and the macrolides were shown to range between 10 to >20 (Johnson et al., 1980; Hand et al., 1984). Fluoroquinolones have also been found to have variable intracellular penetration properties: the concentrations of moxifloxacin (MXF) have been shown to be 20–70 fold higher in pulmonary alveolar macrophages when compared to the concentrations in the plasma (Soman et al., 1999). The concentrations of levofloxacin were only marginally higher in alveolar cells when compared with concentrations in the plasma (Andrews et al., 1997). On the other hand, ciprofloxacin was found to have an alveolar macrophage to plasma ratio of 5–10, indicating an intermediate penetration capacity (Schüler et al., 1997).

The penetration and accumulation of TB drugs inside *Mtb* is the final step of the long journey the drugs have to take to reach the site of action. The reduced permeability of *Mtb* to small-molecule drugs has also been shown to contribute to the drug tolerance found in the dormant *Mtb* population (Dartois, 2014). Nutrient-starved non-replicating *Mtb* also display a significant reduction in intracellular accumulation of fluoroquinolones, which may help explain the resistence of quiescent *Mtb* against fluoroquinolone activity (Sarathy et al., 2013a). Polyamines decrease the ability of drugs to permeate *Mtb*, and have also been shown to prevent the uptake of fluoroquinolones by the mycobacteria (Sarathy et al., 2013b). These results, therefore suggest that the intracellular production of polyamines by macrophages might be partially responsible for the dormancy and phenotypic drug resistance of intracellular *Mtb* (Dartois, 2014).

There is therefore an urgent need to develop new therapeutic approaches for the treatment of TB (Kimmey and Stallings, 2016; Kaufmann et al., 2018; Paik et al., 2019). Host directed therapy (HDT) is a novel and promising concept in TB treatment, where small molecules, together with or without additional antibodies, are used to modify host responsesto better control the progression of TB. HDT drugs are distinct from antibiotics, as they directly modulate the functions of host cells without interacting with *Mtb*; thereby preventing the development of drug resistance by the infecting pathogen (Kolloli and Subbian, 2017; Paik et al., 2019). The applications of HDT-TB can possibly be effective in the treatment of MDR or XDR-TB through the therapeutic targeting of various clinically relevant biological pathways in hosts. Modulation of pathological or protective responses in hosts has revealed that various components of the immune system are central therapeutic targets for HDT against TB (Zumla et al., 2016; Kolloli and Subbian, 2017; Paik et al., 2019).

Autophagy is an intracellular catabolic process that assists in the maintainance of homeostasis or the removal of invading pathogens through a lysosomal degradation mechanism. The activation of autophagy in infected host cells, through the use of various drugs/compounds is a promising treatment strategy against *Mtb* infection, which may also work against drug-resistant strains with or without adjunctive agents (Gupta et al., 2016; Kimmey and Stallings, 2016; Paik et al., 2019). As autophagy and HDT are still relatively new approaches in TB, there is a scarcity of research focusing on the delivery of autophagy inducing compounds (AICs) and the treatment duration of autophagy therapy aspecially when combined with conventional antibiotic treatment. However, it is still necessary to directly deliver the AICs to the infected cells/tissues/organs to prevent unwanted side effects and delivery to non-target cells (Paik et al., 2019).

Nanoparticles (NPs) can be utilized as a delivery system to improve the activity of AICs against intracellular *Mtb* by transporting the encapsulated AICs to their target sites while protecting them from biodegradation and enhancing their absorption across biological barriers (Dube et al., 2013; Tukulula et al., 2018). NPs also allow for sustained drug release in the target tissue. This allows for the reduction of dosing frequency, thus lessening the drug-associated side effects in the process (Grabowski et al., 2013). In addition, the materials used to synthesize the NPs can possess autophagy inducing activity or the surface of the NPs can be functionalized with an AIC (Bekale et al., 2018).

In this review, we focus on the macrophage responses to *Mtb* infection, in particular, the mechanistic aspects of autophagy and the evasion of autophagy by intracellular *Mtb.* Due to the overlap between the onset of autophagy and apoptosis; we also focus on the relationship between apoptosis and autophagy. We will also review known AICs in the context of *Mtb* infection. Finally, we discuss the applications of NPs in inducing autophagy with the intention of sharing insights to encourage further research and development of nanomedicine HDTs for the treatment of TB.

## Uptake of *Mtb* and Fate Within Macrophages

TB can be spread when people with active pulmonary TB expel *Mtb* into the air, for example, by coughing. Aerosols of the pathogen can persist in the air for a prolonged period of time and once inhaled, the bacilli travel through the upper respiratory tract to reach the alveoli in the lungs, where they are taken-up primarily by alveolar macrophages, type 2 pneumocytes and polymorphonuclear neutrophils (PMNs). However, alveolar macrophages commonly act as the first line of defense against the pathogen (Silva Miranda et al., 2012; Dartois, 2014; Bekale et al., 2018). *Mtb* is internalized by macrophages through phagocytosis and subsequently located within phagosomes, which undergoa series of fusion events to mature and attain anti-microbial properties. The progression of phagosome maturation is actively regulated and driven by the network of Rab GTPases (Rab), which also play a role in the identity of the endosomal organelle (e.g., Rab5, early endosomes; Rab7, late endosome), sorting of protein and lipids through the recycling pathway as well as regulating membrane-fusion events (Gruenberg and van der Goot, 2006; Gutierrez, 2013; Prashar et al., 2017).

Therefore, the process of phagosome maturation is characterized by the recruitment of specific Rab GTPases, which ultimately facilitate the fusion between the phagosome and the lysosome to form the phagolysosome, a cytoplasmic body that contains a set of hydrolytic enzymes necessary for the clearance of pathogens. Rab20, Rab34 and the proneurotropin receptor sortilin have also been described as important regulators of phagosome maturation and are thus critical in the control and eradication of intracellular *Mtb* (Kasmapour et al., 2012; Vazquez et al., 2016; Schnettger et al., 2017). The completion of phagosome maturation is accompanied by a reduction in intraphagosomal pH from neutral to the more acidic pH 5, *via* a vesicular proton-pump ATPase (HC-V-ATPase)-dependent process (Russell et al., 2009). Phagosomal acidification creates a suitable environment for the production of reactive oxygen species (ROS) and for the optimal activity of lysosomal digestive enzymes; hence, the process is essential for the clearance of intracellular *Mtb* (Vieira et al., 2002; Sun-Wada et al., 2009).

## Surface Recognition and Macrophage Responses to *Mtb* Infection

The uptake of *Mtb* by phagocytic macrophages is facilitated by the recognition of Pathogen Associated Molecular Patterns (PAMP) present at the bacterial surface by various Pattern Recognition Receptors (PRRs) on the surface of macrophages such as Scavenger Receptors (SR), cytosolic DNA sensors,C-type Lectin Receptors (CLR/CTL), Fc Receptors (FcR) and Toll-Like Receptors (TLR), mannose receptors, surfactant protein A receptors, CD14, immunoglobulin receptors, and complement receptors (Songane et al., 2012; Nasiruddin et al., 2017; Queval et al., 2017). Phagocytosis, innate immune responses and various cellular processes such as antigen presentation, inflammasome activation, autophagy, phagosome maturation, and apoptosis are dependent on the stimulation of the PRRs (Queval et al., 2017). *Mtb* has been shown to activate phagocytosis in macrophages by interacting with TLRs. Recognition of *Mtb* is more specifically facilitated by TLR2 using a variety of microbial structures, such as the lipoproteins19 kDa, lipomannans (LM), lipoarabinomannan (LAM) and the glycolipid phosphatidyl-*myo*-inositolmannoside (PIM) (Quesniaux et al., 2004).

It has been noted that the loss of lipooligosaccharide (LOS) production in the evolution of TB causing mycobacteria is responsible for the rough colony morphology of these pathogens, further contributing to enhancing the recognition of *Mtb* by TLR2 (Boritsch et al., 2016). In addition, TLR9 has been reported to recognize unmethylated CpG motifs in bacterial DNA (Bafica et al., 2005). These events trigger a signaling cascade that stimulates Myeloid Differentiation primary response protein 88 (MyD88) resulting in the activation of pro-inflammatory cytokine production, activation and nuclear translocation of transcription factors, including nuclear factor kappa-light-chain-enhancer of activated B cells (NF-kB) (Nigou et al., 2001).

C-type lectin receptors (CLR/CTL) are a family of calcium dependent membrane bound receptors that recognize carbohydrate rich molecules. Among these receptors, the mannose Receptor (MR) is one of the most well-known, and recognizes mannose on the surface of *Mtb*. The stimulation of macrophages through the MR has been shown to induce the production of anti-inflammatory cytokines without activating oxidative responses (Nigou et al., 2001). The Dendritic Cell immune Activating Receptor (DCAR), Dectin-1, Dectin-2, C-type lectin superfamily member 8 (Clecsf8) and the Mincle receptor belong to a sub-family of the CLR and represent the most known CLR expressed by macrophages (Yonekawa et al., 2014; Marakalala and Ndlovu, 2017).

DCAR is an Fc receptor γ-chain (FcRγ) coupled receptor that induces Th1 responses against *Mtb* infections by recognizing PIM. T cells of DCAR-deficient mice have been shown to have weakened IFN-γ production resulting in elevated bacterial loads (Marakalala and Ndlovu, 2017). Dectin-1 is a transmembrane receptor with an extracellular C-type lectin-like domain (CRD) and a cytoplasmic immunoreceptor tyrosine-based activation motif (ITAM)-like domain (hemITAM). Dectin-1 is a known fungal β (1, 3)-glucan receptor but the precise PAMP for *Mtb* remains unknown. However, Dectin-1 triggers the production of IL-12p40 in a Syk-dependent mannerin splenic DCs infected with *Mycobacterium Bovis* (*M*. *bovis)* or pathogenic *Mtb* (Dinadayala et al., 2004; van de Veerdonk et al., 2010; Marakalala and Ndlovu, 2017).

Dectin-2 on the other hand, has been shown to recognize ManLAM to induce host immune responses against *Mtb* infection. The structure of Dectin-2 consists of a transmembrane domain, a CRD, and a short cytoplasmic tail. The short tail recruits an ITAM-linked FcRγ, which activates signaling through Syk and possibly the Caspase recruitment domain family member 9/B-Cell CLL/lymphoma 10/Mucosa-associated lymphoid tissue lymphoma translocation protein 1 (CARD9/BCL10/MALT1) complex. Dectin-2 has also been shown to recognize the virulent *Mtb* H37Rv strain, however, the protective capacity of this receptor against *Mtb* is yet to be demonstrated *in vivo* (Toyonaga et al., 2016; Marakalala and Ndlovu, 2017).

The Mincle receptor recognizes and specifically binds to what is thought to be likely the most abundant glycolipid in the mycobacterial cell wall, the mycobacterial cord factor Trehalose-6,6-dimycolate (TDM) (Ishikawa et al., 2009; Marakalala and Ndlovu, 2017). The ligation of TDM to Mincle triggers the generation of Th1/Th17 immune responses, production of pro-inflammatory cytokines and granuloma formation (Ishikawa et al., 2009; Marakalala and Ndlovu, 2017; Mishra et al., 2017). Clecsf8 also interacts with mycobacteria through the TDM glycolipid. This PRR is commonly expressed on macrophages, classical monocytes, peripheral blood neutrophils, and by a subsets of dendritic cells (DCs). Upon engaging with TDM, Clecsf8 stimulates Mincle expression through a protein to protein interaction, resulting in an increase in intracellular responses. Downstream signaling of this FcRγ-coupled receptor is mediated through Syk kinase and CARD9/BCL10/MALT1 pathways and induces various intracellular responses, including NF-κβ activation, pro-inflammatory cytokine production, phagocytosis, and respiratory burst. Clecsf8 interaction with TDM also induces DC maturation and T-cell priming (Marakalala and Ndlovu, 2017).

Complement Receptor (CR) and Fc receptors are also highly expressed on the surface of macrophages and CR3 has been shown to help facilitate the phagocytosis of *Mtb* by macrophages through the recognition of PIM and/or *Mtb* polysaccharides (Villeneuve et al., 2005). Mammalian monocytes and macrophages express scavenger receptors to recognize oxidized or acetylated lipoproteins. The Macrophage Receptor with Collagenous (MARCO) structure is the most studied scavenger receptor and has been found to cooperate with TLR2 to stimulate the secretion of proinflammatory cytokines and the activation of transcriptional factor NF-kB through the recognition of TDM (Bowdish et al., 2009). DCs and macrophages use Dendritic Cell Specific Intercellular adhesion molecule-3 Grabbing Non-integrin (DCSIGN/CD209) to recognize conserved sugar motifs on various parasites, viruses, and bacteria, including *Mtb* (Tanne and Neyrolles, 2010).

## Cell Death in *Mtb* Infected Macrophages

The failure to eradicate intracellular *Mtb* post-infection, results in either the active or latent form of TB. In the latent phase, an assortment of immune cells (more macrophages, lymphocytes and DCs) are recruited to the region of infection to keep the intracellular bacteria inactive. Together these cells may at times form a tissue structure known as a granuloma, which consists of a core composed of *Mtb* infected macrophages. In the presence of activated T cells, the granuloma becomes fully organized and the core becomes enclosed with lymphocytes (mainly γ/δ T Cells, CD4^+^ and CD8^+^) which initiate the secretion of IL-8, IL-12, TNF-α, and other pro-inflammatory cytokines by macrophages (Ehlers and Schaible, 2012; Hajipour et al., 2012; Nasiruddin et al., 2017). The end result consists of a continuous balance between necrotic infected cells and the recruitement of new phagocytes onto the granuloma is known as latency and is one of the hallmarks of latent TB (van Crevel et al., 2002; Hajipour et al., 2012; Nasiruddin et al., 2017).

**Figure 1:**
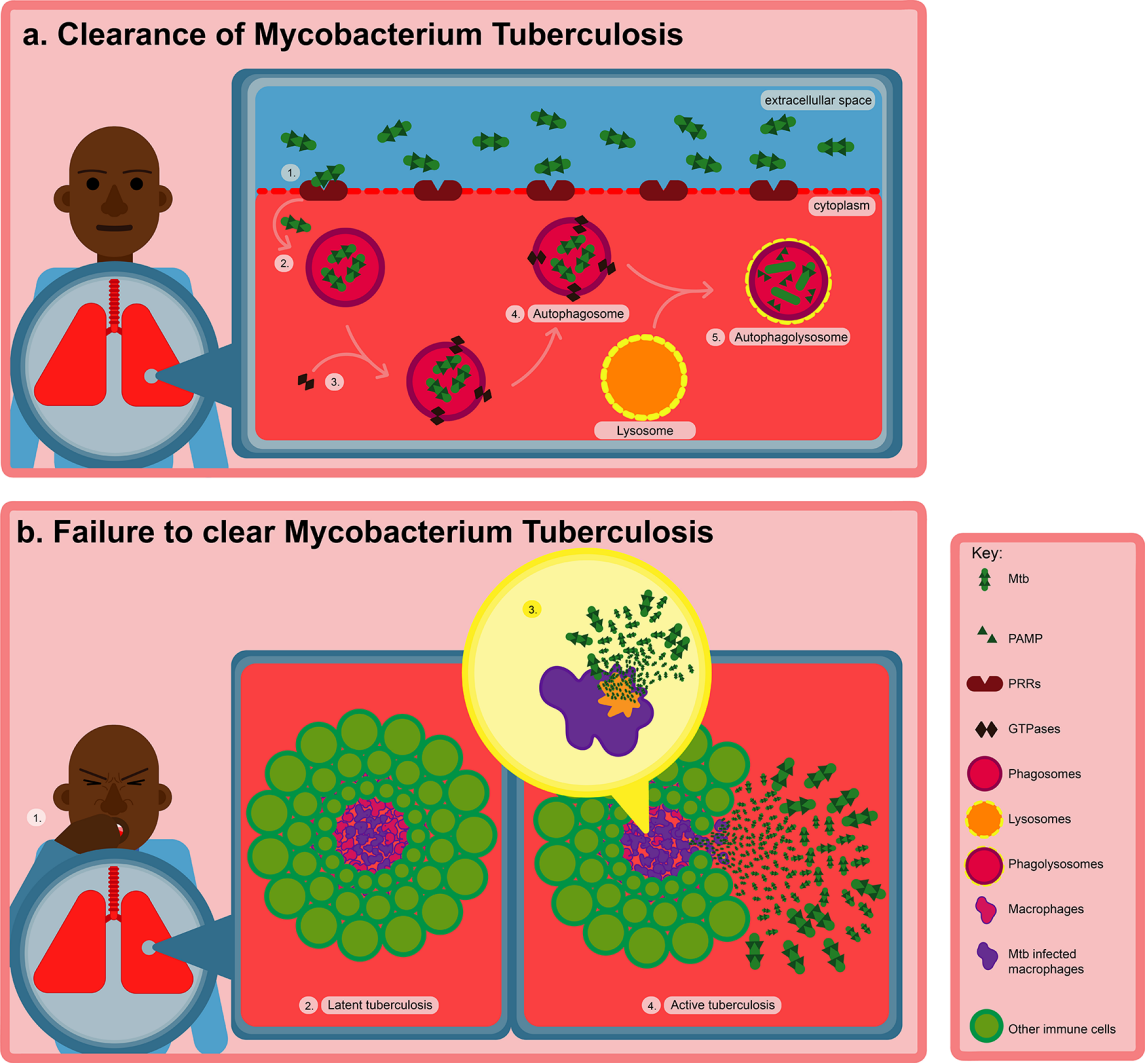
Transmission and pathogenesis of TB after *Mtb* infection. **(A)** Macrophages use PRRs to take up *Mtb* by recognizing various PAMPs on the surface of the bacteria. Recognition of *Mtb* is more precisely facilitated by TLR2, which triggers phagocytosis, innate immune responses and various cellular processes such as antigen presentation, inflammasome activation, autophagy, and apoptosis resulting in the eradication of intracellular *Mtb*. **(B)** However, failure to eradicate intracellular *Mtb* after infection, results in either the latent or active form of TB. In the latent phase, the *Mtb* can remain dormant within the granuloma for years. However, when the immune system becomes weakened, *Mtb* can become reactivated, subsequently inducing the death of the infected macrophages through necrosis.

However, this impasse between the host and pathogen, is dynamic and consists of an equilibrium between cell death and replenishment through cellular recruitment, as well as vascular and tissue remodeling (Ehlers and Schaible, 2012). The bacteria can persist within the granuloma in a latent state for prolonged periods of time (lasting up to the lifetime of the individual), however, as illustrated in **Figure 1**, a weakened immune system (e.g., due to malnutrition, HIV infection etc.) can lead to the reactivation of the *Mtb*. In this active phase, the bacteria can begin to proliferate, ultimately inciting the death of the infected macrophages through necrosis (Silva Miranda et al., 2012). *Mtb* can now exit the granuloma and further disseminate to form lesions in other regions of the lungs. The active *Mtb* can also be transmitted to other individuals through for example, coughing (Mueller and Pieters, 2006).

**Figure 2:**
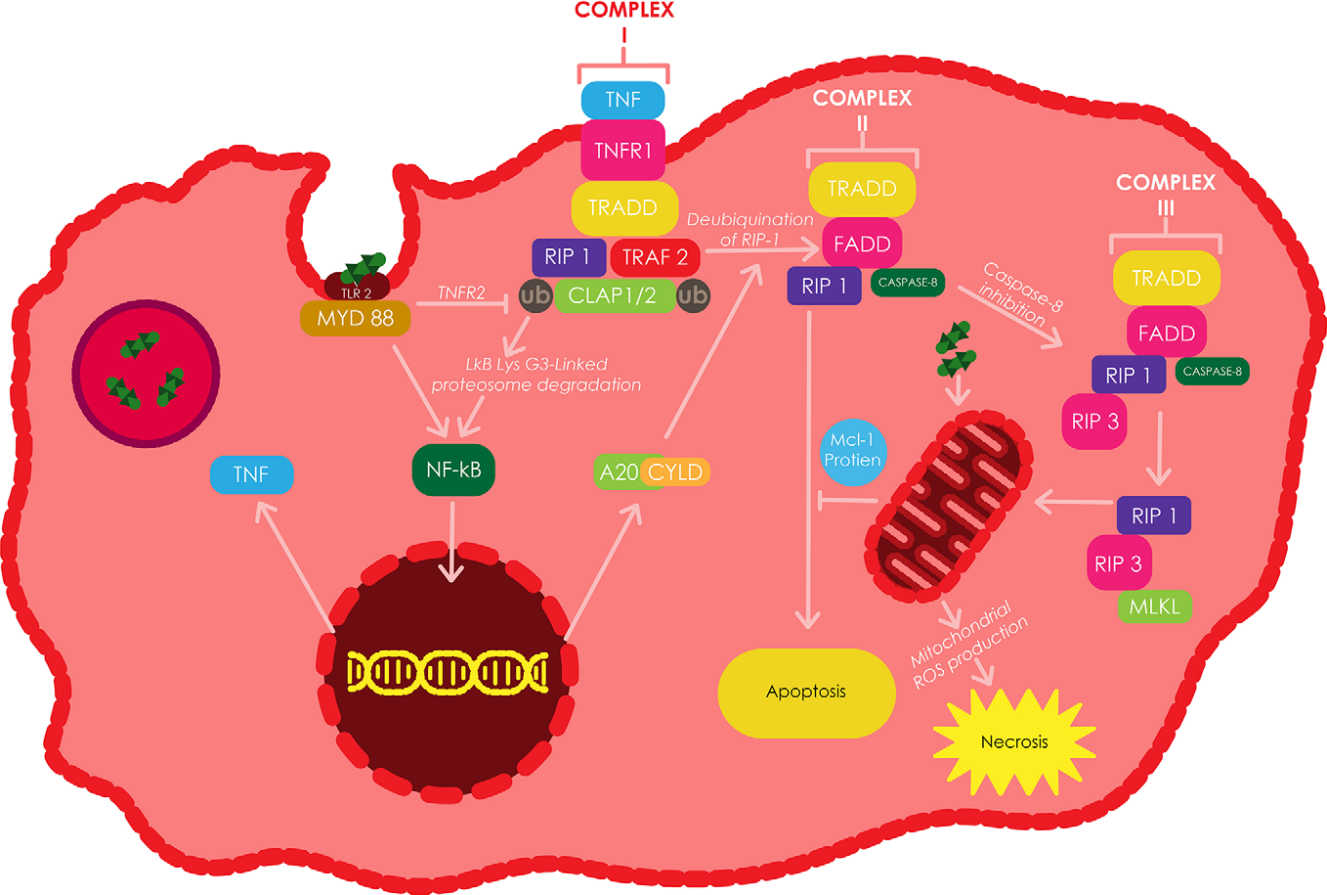
The induction of apoptosis and/or necroptosis by TNF in *Mtb* infected macrophages. TLR2 can be stimulated by different components of the *Mtb* cell wall, which triggers the translocation of NF-κB to the nucleus resulting in the silmultaneous expression of TNF through a myeloid differentiation factor 88 (MyD88) dependent approach. The binding of TNF to TNFR1, induces the binding of TRADD to RIP1 and TRAF2/5, which consecutively bind to cIAP1/2 to make up complex I. cIAPs facilitates the Lys-63-linked polyubiquitination of RIP1 or TRAF2 resulting in Lys 63-linked proteasomal degradation of IκB. The resulting NF-κB translocates into the nucleus and triggers the transcription of various genes including A20 and CYLD. RIP1 becomes deubiquitinated by A20 and CYLD, futher switching complex I into complex II, which then initiates apoptosis through the recruitment and activation of Caspase-8. Casepase 8 inactivation triggers the formation of complex III, which induces the recruitment of RIP3 by RIP1 and subsequently the recruitment of MLKL by RIP3, ultimately resulting in necroptosis. Adapted from (Xu et al., 2014).

Macrophages infected with *Mtb* commonly express two types of cell death, i.e., necrosis: a death modality that ends in cell lysis and apoptosis: a form of cell death that maintains an intact cell membrane (**Figure 2**) (Behar et al., 2011). Apoptosis can be instigated through various mechanisms; nonetheless, the process is defined using several molecular and morphological decisive factors. The common hallmarks of apoptosis are DNA fragmantation, exposure of phosphatidylserine on the outer leaflet of the plasma membrane, and lastly, containment of cellular components in membrane-bound blebs (Behar et al., 2011).

## The Intrinsic Pathway: The Role of the Mitochondria in the Induction of Apoptosis and Necrosis

Vertebrate cells generally undergo apoptosis by utilizing the intrinsic apoptotic pathway; relying on mitochondrial outer membrane permeabilization (MOMP) as the defining hallmark (Green and Kroemer, 2004). MOMP facilitates the release of apoptosis inducing factor, Smac-DIABLO, cytochrome *c*, and other factors from the mitochondrial intermembrane resulting in the activation of caspase-3/7 which are responsible for the induction of apoptosis. These events can also occur due to the opening of the mitochondrial inner membrane pore (permeability transition pore), resulting in the loss of the mitochondrial intermembrane potential (Δ*ψ*m), mitochondrial permeability transition (MPT), and ultimately the induction of necrosis. *Mtb* infections have also been shown to induce these changes in mitochondrial membranes; therefore, these changes are believed to determine the mode of death in infected macrophages (Behar et al., 2011).

In the first scenario, MPT can cause the mitochondria to become permeable to water, resulting in swelling, dysfunction, and ultimately necrosis (Green and Kroemer, 2004). In the second scenario, MOMP can occur as a by-product of the outer mitochondrial membrane damage caused by irreversible MPT. Such a scenario can materialize when hepatocytes undergo apoptosis and/or necrosis due to oxidative stress, or other toxic treatments (Kim et al., 2003). However, the occurance of apoptosis and MOMP is not always dependent on MPT. In this scenario, members of the Bcl-2 family of apoptosis inducing proteins, for example, induce MOMP without affecting the mitochondrial inner membrane (Chipuk and Green, 2008). More specifically, processed Bcl-2 only protein BID activates the proapoptotic Bcl-2 family proteins BAX and BAK which subsequently induce MOMP and translocation of proapoptotic factorsinto the cytosol, resulting in the activation of caspase-9, caspase-3 and ultimately apoptosis (Behar et al., 2011).

A permeable mitochondrial outer membrane allows for effector molecules to damage the mitochondrial inner membrane by gaining access into the mitochondrial intermembrane space. This could be the same mechanism utilized by capase-3 to damage a component of the electron transport chain called Ndufs1 (Ricci et al., 2004). Ndufs1 damage induces ROS production by disrupting the electron transport chain in the inner membrane, resulting in necrosis. It is believed that caspase-3 gains access to the mitochondrial intermembrane space through a permeable mitochondrial outer membrane, which also facilitates the escape of proapoptotic factors including cytochrome *c* into the cytosol (Behar et al., 2011). Finally, there is a scenario where MPT can occur independently from the induction of MOMP. This is believed to be a mechanism utilized by the protease, granzyme A, to damage the components of the mitochondrial inner membrane (Martinvalet et al., 2008). Granzyme A enters the mitochondrial intermembrane space without damaging the mitochondrial outer membrane, due to the cytosolic chaperone activity of Hsp70 and Hsp90. Once inside, the protease cleaves Ndufs3 resulting in MPT (Martinvalet et al., 2008).

## Induction of Apoptosis Through the Extrinsic Pathway in *Mtb* Infected Macrophages

Various components of the mycobacterial cell wallcan stimulate the production of tumor necrosis factor (TNF) in *Mtb* infected macrophages through the TLR2-mediated signaling pathway. TNF induces apoptosis through TNF receptors, which can be found as either membrane-bound or in soluble form (Xu et al., 2014). TNF minimizes the spread of *Mtb* by activating the Caspase-8-mediated extrinsic cell death pathway (Behar et al., 2011). Avirulent or attenuated *Mtb* strains such as *Mtb* H37Ra have been shown to induce more macrophage apoptosis when compared to virulent strains such as *Mtb* H37Rv, which have been confirmed to elude TNF-dependent apoptosis by activating the release of membrane-bound TNFR2 in solution, through an IL-10 dependent pathway (Behar et al., 2011), and by increasing the expression of Mcl-1 protein, a member of the anti-apoptotic B-cell lymphoma/leukemia-2 (Bcl-2) family of proteins which are located in the outer membrane of mitochondria (Xu et al., 2014).

The virulent strain H37Rv has been shown to induce an enhanced release of IL-10 in *Mtb* infected macrophages, when compared to the attenuated strain H37Ra. This elevated IL-10 release triggers a greater build up of sTNFR2, which through the formation of a soluble receptor-ligand complex leads to the inhibition of TNF-α activity (Balcewicz-Sablinska et al., 1998). Conversely, increased production of TNF has been shown to induce mitochondrial ROS in infected macrophages *via* the receptor interacting protein 1 (RIP1) receptor interacting protein 3 (RIP3) mixed lineage kinase domain-like protein (MLKL) dependent pathways. The mitochondrial ROS induces a type of necrosis called necroptosis in *Mtb* infected macrophages, which leads to the release of mycobacteria into the extracellular environment (Wang et al., 2012). The repression of RIP3 or MLKL has been shown to switch *Mtb* infected macrophages from a state of TNF-induced necroptosis to a state of delayed RIP1-dependent apoptosis (Han et al., 2011). The stimulation of TNF receptor 1 (TNFR1) by TNF triggers the binding of TRADD (TNF receptor-associated death domain) to RIP1, TNF receptor-associated factor 2/5 (TRAF2/5) and cIAP1/2 (cellular inhibitor of apoptosis 1/2) to form complex 1 (Xu et al., 2014).

The Lys 63-linked polyubiquitination of RIP1 or TRAF2 by cIAPs induces the translocation of NF-κB into the nucleus to induce the transcription of A20 and cylindromatosis (CYLD), which can both deubiquitinate RIP1 (Vandenabeele et al., 2010). Inhibition of cIAP proteins or deubiquitination of RIP1 facilitates the conversion of complex I to complex II, which is comprised of FADD, RIP1, TRADD and Caspase-8 (Han et al., 2011). Complex II facilitates the activation of Caspase-8 which further instigates apoptosis. The inhibition of Caspase-8 activity triggers the recruitment of RIP3 by RIP1 to form complex III (also called necrosome), which also comprises of Caspase-8, FADD and possibly TRADD. RIP1 subsequently phosphorylates RIP3 to recruits MLKL, resulting in the onset of necroptosis. In addition, the presence of inhibitors of ubiquitinating enzymes, virulent strains of *Mtb*, and deubquitinating enzymes keep Caspase-8 inactive (Xu et al., 2014).

Therefore, avirulent *Mtb* strains tend to stimulate apoptosis, whereas virulent *Mtb* strains are inclined to trigger necroptosis, a type of cell death known to favourthe pathogen and facilitate the dissemination of *Mtb* (Jayaraman et al., 2013). These observations suggest that at some point during the infection, virulent *Mtb* strains stimulate higher expression of TNF when compared to avirulent strains resulting in necroptosis. In addition, virulent strains possibly secrete a biological agent that blocks the activity of Caspase-8, reslting in the inhibition of apoptosis. In contrast, avirulent strains might be lack this ability (Xu et al., 2014).

Macrophage apoptosis is associated with an increase in ROS production. During apoptosis, DCs engulf and facilitate the degradation of the *Mtb* or *Mtb* product containing apoptotic bodies by cross-presenting the bacterial antigens to CD8^+^ T cells through a process called efferocytosis (Lee et al., 2004; Ashida et al., 2011; Silva Miranda et al., 2012). The binding of apoptotic bodies to the phagocytes during efferocytosis triggers the expression of anti-inflammatory cytokines such as interleukin-10 (IL-10) and transforming growth factor-β. The suppression of inflammation by these cytokines is believed to assist in minimizing the tissue damage caused by the accidental release of intracellular contents, including degradative enzymes, to the extracellular space (Lee et al., 2009). As the bacteria are typically killed during this process, *Mtb* infected macrophages have ‘alternative activation’ which is characterized by the arrest of phagosome maturation, increased secretion of macrophage attractant chemokines and M2 cytokines, suppression of microautophagy and apoptosis inhibition (Lancellotti et al., 2006; Ashida et al., 2011).

Highly virulent *Mtb* strains are known to primarily induce necrosis; however, apoptosis has also been detected in macrophages infected with virulent *Mtb* (**Figure 2**). The induction of apoptosis in *Mtb* infected macrophages has been shown to be TNF-α dependent, although, the virulent *Mtb* strains induce extensively less apoptosis in host cells when compared to related attenuated strains (van Crevel et al., 2002; Behar et al., 2011). This difference in apoptosis induction has been explained using selective induction and release of neutralizing soluble TNF-α receptors by pathogenic strains. In turn, the release of TNF-α receptors is regulated by IL-10 production. Therefore, virulent strains of *Mtb* may selectively induce the production of IL-10, resulting inreduced TNF-α activity and decreased apoptosis of infected cells (van Crevel et al., 2002). However, it is still not clear whether virulent *Mtb* inhibits the induction of apoptosis or prevents its completion by locking its downstream events (Behar et al., 2011).

The TLR2 dependent activation of the NF-kB cell survival pathway and the enhanced production of the soluble TNF receptor 2 (sTNFR2), neutralize the activity of TNFR1 resulting in *Mtb* persisting inside the macrophage phagosomes in either an active form or a dormant form (Lancellotti et al., 2006; Ashida et al., 2011). Therefore, in order to investigate the role of apoptotic macrophages in adaptive immunity, a new adoptive transfer model has been developed where *in vitro Mtb* infected macrophages have been transferred from wild type or knockout mice and instilled into normal recipient mice *via* the trachea. This approach has been used to study the influence of macrophage genotypes on the T-cell response and control of infection (Divangahi et al., 2010).

## Role of Eicosanoids in Mitochondrial Protection

**Figure 3:**
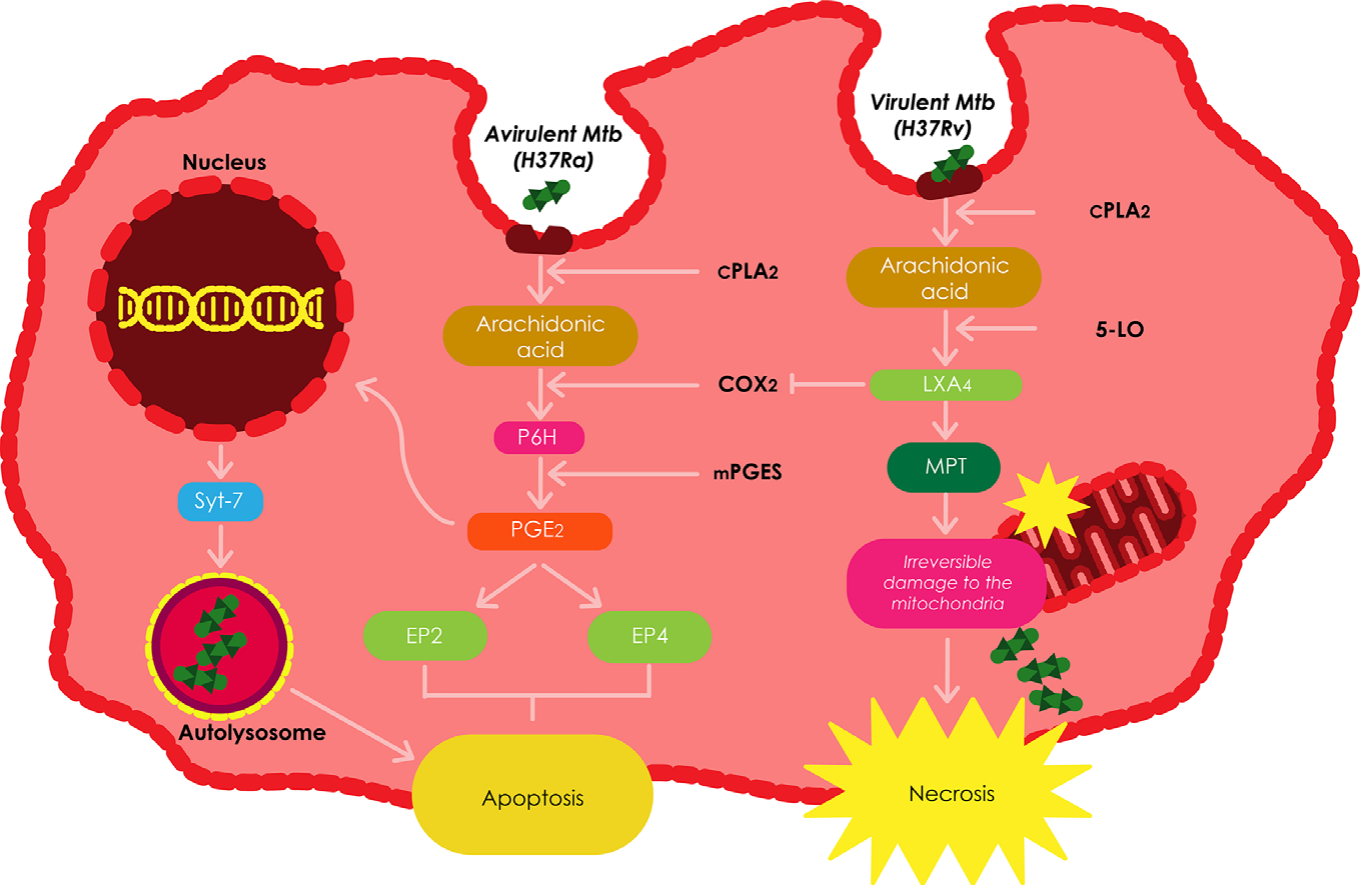
The roles played by eicosanoids and lipoxins in regulating the death of infected macrophages. The balance between prostanoid and lipoxin production is the dictator of the modality of death in *Mtb* infected macrophages. Increased production of LXA_4_ induces necrosis; whereas, excess production of PGE_2_ inhibits necrosis and favors apoptosis. Adapted from (Behar et al., 2011).

Recent studies have investigatedhow the eicosanoid prostaglandin (PGE_2_) and lipoxins (LXs) regulate programmed celldeath in *Mtb* infected macrophages (**Figure 3**). Cyclooxygenases (COX), namely COX1 and COX2, are responsible for the production of PGs by metabolizing arachidonic acid into PGH (Kurtz et al., 2006; Behar et al., 2011). The various PGs produced comprise of PGD_2_, PGE_2_, PGF_2_α, PGI_2_, and thromboxane. Among these, PGE_2_ is the most studied PG, and has a variety of biological functions triggered by interacting withany of its four specific receptors EP1, EP2, EP3, and EP4 (Kurtz et al., 2006; Behar et al., 2011). The interaction between PGE_2_ and the EP2 receptor prevents bacteria induced damage to the mitochondria in macrophages. On the contrary, interacting with EP4 triggers the repair of the plasma membrane in infected macrophages through the recruitment of lysosomal membranes to the cell surface (Behar et al., 2011).

*Mtb* H37Ra has been shown to stimulate PGE_2_ in host macrophages; however, virulent *Mtb* has been demonstrated to stimulate the production of LXA_4_in macrophages (Bousso, 2008). In these cells, 5-lipoxygenase (5-LO) metabolizes arachidonic acid to produce LXA_4._ The ensuing LXA_4_ plays a key role in the onset of inflammation and has been shown to stimulate macrophages to engulf apoptotic neutrophils using phagocytosis (Behar et al., 2011). LXA_4_ inhibitsthe expression of COX2 and thus, the generation of PGE_2,_ subsequently redirecting the fate of the infected macrophage to necrosis. Other pro-necrotic roles of LXA_4_ include inhibiting the stimulation ofapoptosis by lipopolysaccharide/interferon-γ, prevention of the secretion of pro-apoptotic mediators by the mitochondria, as well as inhibiting caspase activation and ROS generation. This data establishes LXA_4_ as the most important mediator of necrosis (Behar et al., 2011).

The fate of the macrophage following *Mtb* infection is a key component of the immune response against the pathogen. Several PGE_2_ dependant cellular pathways have been found to promote apoptosis while preventing necrosis in *Mtb* infected macrophages (Bousso, 2008). These pathways include the induction of prostaglandin H (PGH) synthase which is propelled by membrane associated prostaglandin E synthase-1 (mPGES) to produce PGE_2_, the production of COX2, transcription of synaptotagmin-7 (Syt-7), a lysosomal Ca ^2+^ sensor that facilitates the fusion of the plasma membrane with lysosomal vesicles requiredto induce apoptosis, prevention of irreversible damage of the mitochondrial inner membrane by virulent *Mtb* strains using MPT (Bousso, 2008), and the production of plasminogen activator inhibitor type 2 (serpin B2) (Winau et al., 2006).

In addition, PGE_2_ also induces the transcription of Syt-7, which facilitates the repair of the plasma membrane repair by means of lysosomal exocytosis (Pozzi et al., 2005). There are numerous PGE_2_ dependent mechanisms leading to apoptosis, and the balance between the production of PGE_2_ and LXA_4_ regulates the number of cells undergoing apoptosis or necrosis (Behar et al., 2011).

## Autophagy as an Innate Immune Defense Mechanism

**Figure 4:**
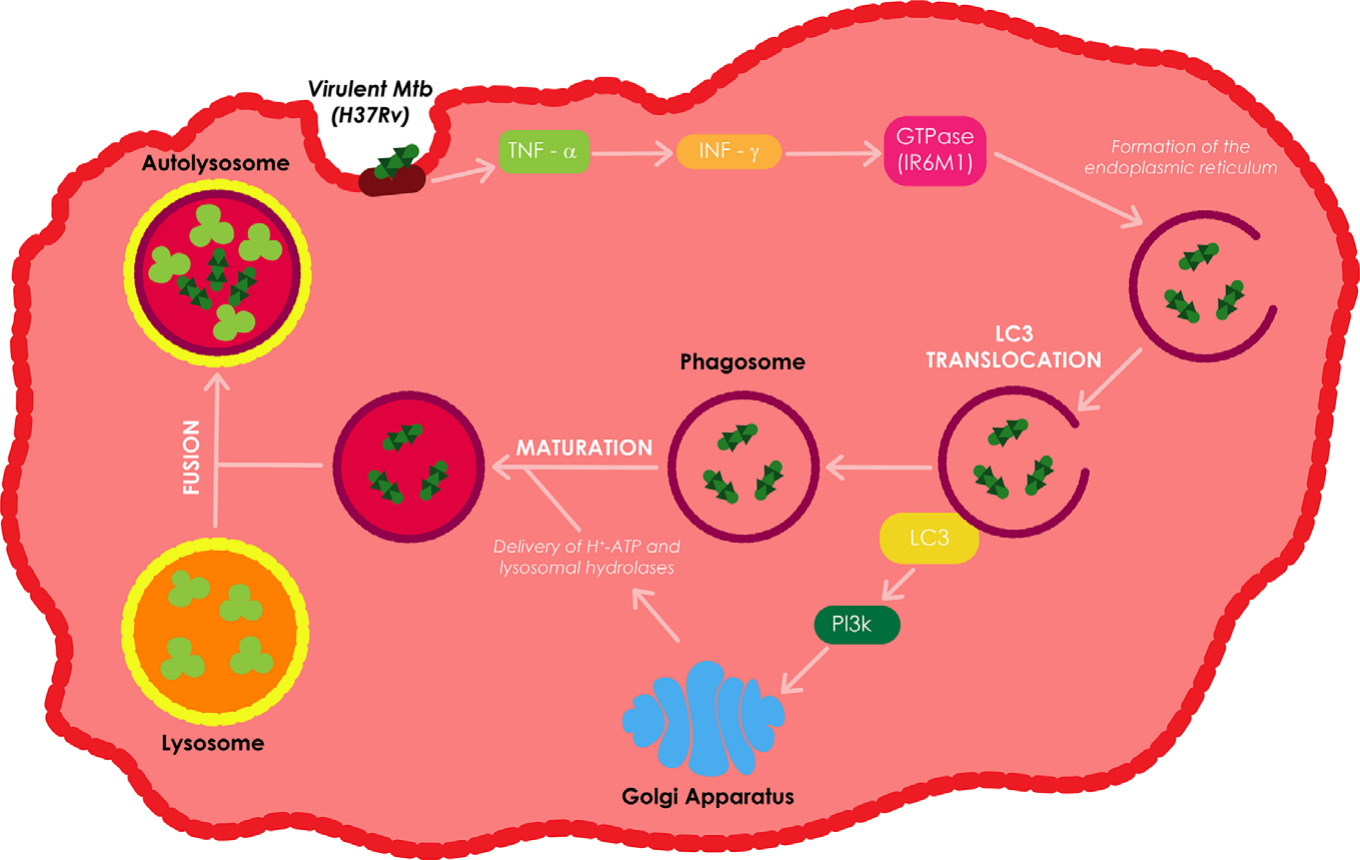
Overview of macroautophagy. Macroautophagy is one of autophagic process which is induced by various stimuli or stress including infection. Autophagy sequesters aggregated proteins, damaged organelles, and microbes from cytoplasm through formation of double-membraned structure (autophagosome). These autophagosomes fuse with lysosome or endosome that contains endocytic compartments to form amphisome or autolysosome. Finally, cargos are degraded in autolysosome for the maintenance of cellular homeostasis. Adapted from (Songane et al., 2012).

Autophagy is a homeostatic lysosomal process that involves the degradation of cellular components to their basic elements as a response to stress conditions (Lam et al., 2017; Paik et al., 2019). Based on how the cargo is targeted and delivered to the lysosomes, the autophagy pathway can be catagorized into three different types of autophagy processes, i.e., macroautophagy, microautophagy, and chaperone-mediated autophagy. Macroautophagy (generally referred to as autophagy) is triggered by numerous stress signals such as damaged intracellular organelles, hypoxia, starvation, and microbial infection (Lam et al., 2017; Paik et al., 2019). As illustrated in **Figure 4**, autophagy is initiated by the formation of the early phagophores (isolation membrane) from the endoplasmic reticulum–mitochondria contact sites, followed by the expansion of the phagophores around the intra-cytoplasmic cargo, and the growth of the phagophores into double membrane autophagosomes. Eventually, the autophagosomes become autolysosomes by coalescing with endosomes/lysosomes to facilitate the degradation of cytoplasmic cargo. Autophagy is also essentialfor the regulation of various immune responses, including: inflammation, innate immunity, and macrophage antibacterial defenses (Lam et al., 2017; Paik et al., 2019).

Bacteria mainly use pathogen induced damage-associated molecular patterns (DAMPs) and PAMPs to induce autophagy in host cells (Deretic et al., 2015; Shibutani et al., 2015). Cell surface recognition and cytosolic sensing of these molecules triggers signaling cascades that stimulatethe rapid and localized recruitment of the autophagy machinery (van der Vaart et al., 2014). Transcriptional factors such as NF-kB and transcription factor EB (TFEB) can further regulate autophagy by promoting the expression of diverse autophagy genes that extend the activation of autophagy (van der Vaart et al., 2014; Pastore et al., 2016).

The IL-12 and IL-18 dependent secretion of interferon gamma (IFN-γ), together with the production of tumor necrosis factor alpha (TNF-α) have all been shown to be vital in the defensive instigation of autophagy against *Mtb* infection (Salgame, 2005). IFN-γ induces autophagy in both human and murine macrophage cell lines; this type of autophagy reduces the survival of intracellular virulent *Mtb* H37Rv. IFN-γ enhances the translocation of LC3 and the proteolysis of long-lived proteins, which are both autophagy markers, and subsequently increases autophagosome formation (Shi and Kehrl, 2010). Upon IFN-γ stimulation, GTPase (IRGM1), an autophagy linked gene downstream of IFN-γ, controls *Mtb* replication through phagosome formation, facilitates the PI3K-dependent maturation of *Mtb* containing phagosomes, and autophagolysosome formation through the fusion of the phagosome to the lysosome (MacMicking et al., 2003).

The delivery of H^+^-ATP and lysosomal hydrolases from the trans-Golgi network to the phagosome as well as the fusion of the phagosome with the lysosome enhances the acidification of the internal environment of the autophagolysosome or autolysosome resulting in the killing of *Mtb* (Fratti et al., 2003; Songane et al., 2012). Treatment of *Mtb* infected macrophages with TNF-α leads to an increase inIFN-γ induced phagosome maturation in these macrophages (Songane et al., 2012). Autophagy can also produce and supply antimicrobial peptides to the bacterial compartment to improve the bacterial killing (Alonso et al., 2007). Depending on the nature and localization of PAMP/DAMP, autophagy can use a mechanism called xenophagy to selectively capture and eradicate bacteria, damaged organelles, and other signaling platforms activated during the infection process (Bah and Vergne, 2017).

## Xenophagy

**Figure 5:**
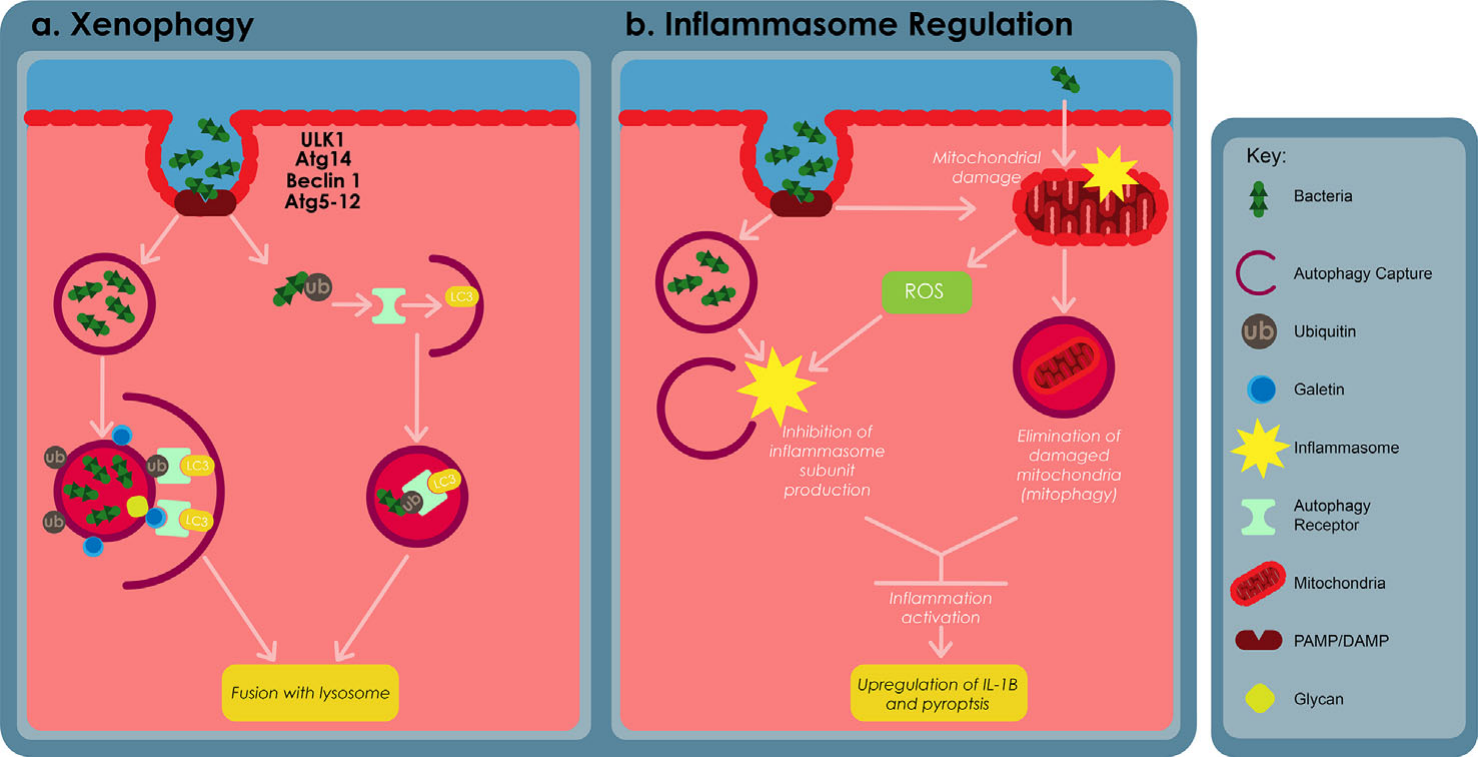
The roles of autophagy in macrophage defences against bacterial infections. **(A)** Cytosolic and vascular pathogens are selectively captured and degraded in the lysosome through a process called xenophagy. Xenophagy depends on ULK1, autophagy related (Atg) 14, Atg5-12, Beclin-1, autophagy receptor proteins, such as p62 and requires formation of an autophagosome. Autophagosomes capture bacterial compartments either through ubiquitination or through host glycan recognition by galectins. **(B)** Autophagy regulates inflammation by restraining inflammasome activation. This can be archieved by inhibiting the production of inflammasome subunits or through the elimination of damaged mitochondria, using a process called mitophagy. Adapted from (Bah and Vergne, 2017).

Xenophagy refers to the process whereby cells use autophagy against pathogens and this cellular mechanism has been found to be an innate component of immune responses. Xenophagy is triggered against *Mtb* infection by the cytoplasmic release of the bacteria through the *Mtb* ESX-1 secretion system (Watson et al., 2012; Paik et al., 2019). The autophagic receptors p62 and NDP52, and the STING dependent cytosolic pathway play a crucial role in the elimination of *Mtb* by xenophagy (Watson et al., 2012; Franco et al., 2017). Mice possessing monocyte derived cells and neutrophils that are devoid of Atg5 have been shown to be more susceptible to *Mtb* infection. However, other Atgs such as Beclin-1 and Atg14 (**Figure 5**), were found to cause no susceptibility. These results therefore suggest that autophagy may not be involved in regulating the initial stages of infection, however these claims are yet to be confirmed (Castillo et al., 2012; Watson et al., 2012; Kimmey et al., 2015).

*Mtb* can also damage the phagosomal membrane and access the cytosol using its ESX-1 secretion system (Watson et al., 2012). Cytosolic sensor c-GAS can recognizethe bacteria’s DNA, thus inducing ubiquitination of the bacteria or its phagosome capture using ubiquitin ligases Parkin and Smurf1 (Watson et al., 2015; Franco et al., 2017). As a result, ubiquitin chains bind to autophagy adaptors, such as p62 and NDP52, recruitmicrotubule-associated-protein-1 light chain 3 (LC3) which subsequently delivers *Mtb* into an autophagosome. Cytosolic lectins of the galectin family also recognize the host glycan present on the lumen of damaged phagosomes, subsequently targeting these damage phagosomes for degradation by means of the autophagosome (Gomes and Dikic, 2014; Chauhan et al., 2015b).

## LC3-Associated Phagocytosis

LC3-associated phagocytosis (LAP) is a non-canonical autophagy process known to play a significant role in antibacterial defenses in macrophages (Galluzzi et al., 2017; Mitchell and Isberg, 2017). During LAP, pathogens are taken up by macrophages through phagocytosis. Binding of pathogens on surface receptors, such as the Fc gamma receptors, toll-like receptors, or Dectin-1, triggers the conjugation of LC3 onto the phagosomal membrane without requiring autophagosome formation (Sanjuan et al., 2007; Huang et al., 2009; Ma et al., 2012). Unlike canonical autophagy, LAP depends on the activation of Beclin-1/Rubicon complex and NADPH oxidase-2 (NOX2) and does not rely on ULK1 or Beclin-1/Atg14 complexes (Huang et al., 2009; Martinez et al., 2015). LC3 conjugation is triggered by the recruitment of two ubiquitin-like conjugation systems that are promoted by phosphatidylinositol-3-phosphate (PI3P) and ROS. The production of ROS has been shown to be PI3P dependent, whereas the synthesis of PI3P relies on Rubicon. The MORN2 protein has also been implicated in the LC3 conjugation pathway, though its role in the process remains unknown (Abnave et al., 2014).

A number of studies have shown that the conjugation of LC3 onto the phagosome improves the fusion between the phagosome and the lysosome. As a result, macrophages with a deficient LAP pathway are less effective at controlling intracellular growth of numerous bacteria including *Mtb*, *Staphylococcus aureus*, *Legionella pneumophila*, *M. bovis* BCG, and *Listeria monocytogenes* (Yang et al., 2012; Abnave et al., 2014). In addition, Rubicon-deficient mice have been shown to retain a larger bacterial load and are more vulnerable to *L. Monocytogenes* infection (Yang et al., 2012). LAP has also been shown to assist in antigen presentation process *via* MHC class II molecules, thus bridging the gap between innate and adaptive immunity (Ma et al., 2012; Romao et al., 2013).

However, it is important to note that LC3 conjugation at times delays or does not affect phagosome maturation. This indicates that LC3 alone is not enough to drive the fusion of the phagosome with the lysosome (Bah and Vergne, 2017). Autophagy can also indirectly regulate phagocytosis by varying the expression of phagocytic surface receptors. For example, suppression of the Atg 7 protein synthesis in macrophages promotes the upregulation of MARCO and MSR1, two class-A scavenger receptors that enable phagocytosis of *M. bovis* BCG and *Mtb* (Bonilla et al., 2013). Autophagy-deficient cells have also been shown to amass p62, thus promoting the dissociation of transcription factor nuclear erythroid-related factor 2 (NFE2L2) from Keap1 and its subsequent translocation into the nucleus, where it facilitates the expression of these receptors. In some cases, the lack of autophagy during infection can lead to reduced phagocytosis dependent on the bacteria (Jacquel et al., 2012).

## Reactive Oxygen Species-Mediated Autophagy Against *Mtb* Infections

**Figure 6:**
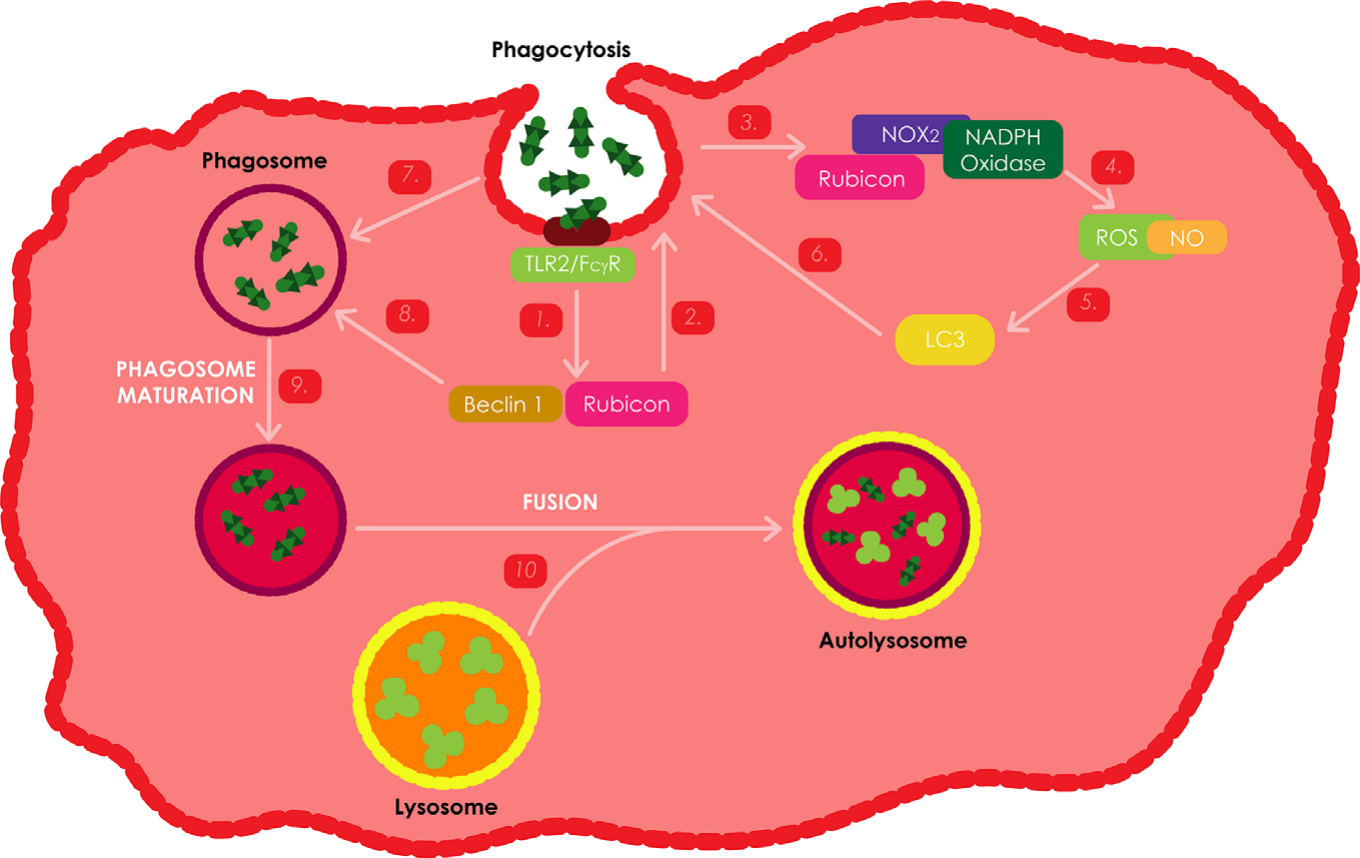
ROS production as an immune response against *Mtb* infections.**** Stimulation of either FcγRs or TLRs during phagocytosis activates similar kinetics to induce theactivation of ROS dependent autophagy. These two receptors activate the NOX2 NADPH oxidase, which is responsible for the production of ROS. Rubicon has also been shown to be an important positive regulator of the NADPH oxidase complex. During *Mtb* infection, the bacteria stimulate TLR2 resulting in the activation of Rubicon to interact with the p22phox subunit of the NADPH oxidase complex, enabling its phagosomal trafficking to induce the production of NOX2 dependent ROS, which leads to the recruitment of LC3 to the phagosome and ultimately the onset of autophagy and phagosome maturation through the assistance of Bectin-1.

ROS serve as signaling molecules that are involved in various cellular processes and play a key role in the killing of intracellular pathogens in macrophages (Lammas et al., 1997). Phagocytosis is a first-line innate immune defense mechanism. As illustrated on **Figure 6**, this process can be activated through the stimulation of specific membrane receptors, such as the β-glucan receptor, complement receptors, Fc_γ_ receptors (Fc_γ_Rs) or TLRs. Previous studies have revealed that the activation of TLR signaling during phagocytosis induces the recruitment of the LC3 autophagy protein to the phagosome, hence promoting phagosome maturation, plausibly by mediating membrane hemifusion/tethering, which results in microbial killing (Huang et al., 2009; Yang et al., 2012). Fc_γ_R and TLR signaling have been shown to induce the assembly of NOX2 NADPH oxidase on the nascent phagosomal membrane, which results in the production of superoxide through the transfer of electrons from the cytosolic NADPH to the oxygen in the phagosome lumen. Superoxide and other ROS generated during this process are highly potent and can directly kill intracellular microbes. The presence of NOX2-generated ROS has also been revealed to be compulsory for targeting LC3 to phagosomes (Huang et al., 2009; Yang et al., 2012).

Upon TLR stimulation or microbial infection (using *Listeria monocytogenes*), Rubicon was found to function as part of a Beclin-1 containing autophagy complex and has been shown to interact with the NADPH oxidase phagocytic complex, resulting in, firstly, the activation of NADPH oxidase which induces ROS-mediated antimicrobial oxidative activity; and secondly, the activation of Beclin-1-kinase complex relocate to phagosomes and trigger phagosomal maturation as a non-oxidative antimicrobial activity (Yang et al., 2012). IFN-γ inducible nitric oxide synthase (iNOS) pathways are an important antimicrobial alternative to the NADPH phagocyte oxidase pathways in phagocytic cells, which facilitates the production of nitric oxide (NO) radicals. Stimulation with zymosan, *L. monocytogenes* and a combination of the two, was found to produce significantly higher levels of ROS and NO in Raw264.7 Rubicon expressing macrophages. However, Rubicon expression as a result of IFN-γ stimulation was found to only induce minor differences in ROS and NO production. These results thus, suggest that Rubicon is primarily involved in the TRL2 dependent antimicrobial activity pathway, and that autophagy can either be ROS and NO dependent or non-dependent (Yang et al., 2012).

## Inflammation Dampening Against Bacterial Infections

Inflammation is crucial to control bacterial infections; however, extreme inflammatory responses (cytokine storm) can result in injured host tissue, thus facilitating the progression of the disease. A number of studies have demonstrated that autophagy regulates inflammation in both infectious and non-infectious conditions (Shibutani et al., 2015; Cadwell, 2016). In cases of infections, autophagy uses multiple mechanisms to down-regulate the activation of inflammasomes (Saitoh and Akira, 2016; Harris et al., 2017). *Pseudomonas aeruginosa* infections damage the mitochondria, resulting in NLRC4 inflammasome. Autophagy eliminates the damaged mitochondria througha process called mitophagy, thus limiting inflammasome activation in both an *in vitro* and *in vivo* setting (Jabir et al., 2015). Atg7 deficient mice have been shown to have enhanced susceptibility to *P. Aeruginosa* infection resulting in increased neutrophil infiltration and severe lung damage. Atg7 deficiency in alveolar macrophages promotes up-regulation of IL-1β and pyroptosis (Pu et al., 2017). Aside frommitophagy, autophagy can also inhibit inflammasome activation by arresting the production of inflammasome subunits (Shi et al., 2012; Kimura et al., 2015).

It has also been shown that the mechanisms of autophagy are greatly manipulated by the micro-environment of the macrophage. Pro-inflammatory cytokines such as IFN-γ, IL-1β, and TNF-α are known to activate autophagy, whereas anti-inflammatory cytokines IL-4, IL-10, and IL-13 seem to inhibit autophagy (Harris, 2011). The recruitment of autophagy proteins p62 and Atg4B also promoted by the IFN-γ mediated xenophagy can only be accomplished through the help of ubiquilin-1 and guanylate binding proteins 1 and 7 (Kim et al., 2011; Sakowski et al., 2015). The presence of vitaminD in serum has been demonstrated to significantly enhance autophagy in macrophages by promoting the expression of cathelicidin, an antimicrobial peptide (Yuk et al., 2009). T cells have also been shownto induce autophagy in *Mtb* infected human macrophages (Petruccioli et al., 2012). In addition, the probiotic, *Bacillus amyloliquefaciens*, has also been shown to up-regulate autophagy genes in macrophages, resulting in enhanced *Escherichia coli (E. coli)* killing (Wu et al., 2017).

## Evasion of Autophagy by Intracellular *Mtb*

### Regulation of Signaling Pathways Involved in Autophagy Initiation

**Figure 7:**
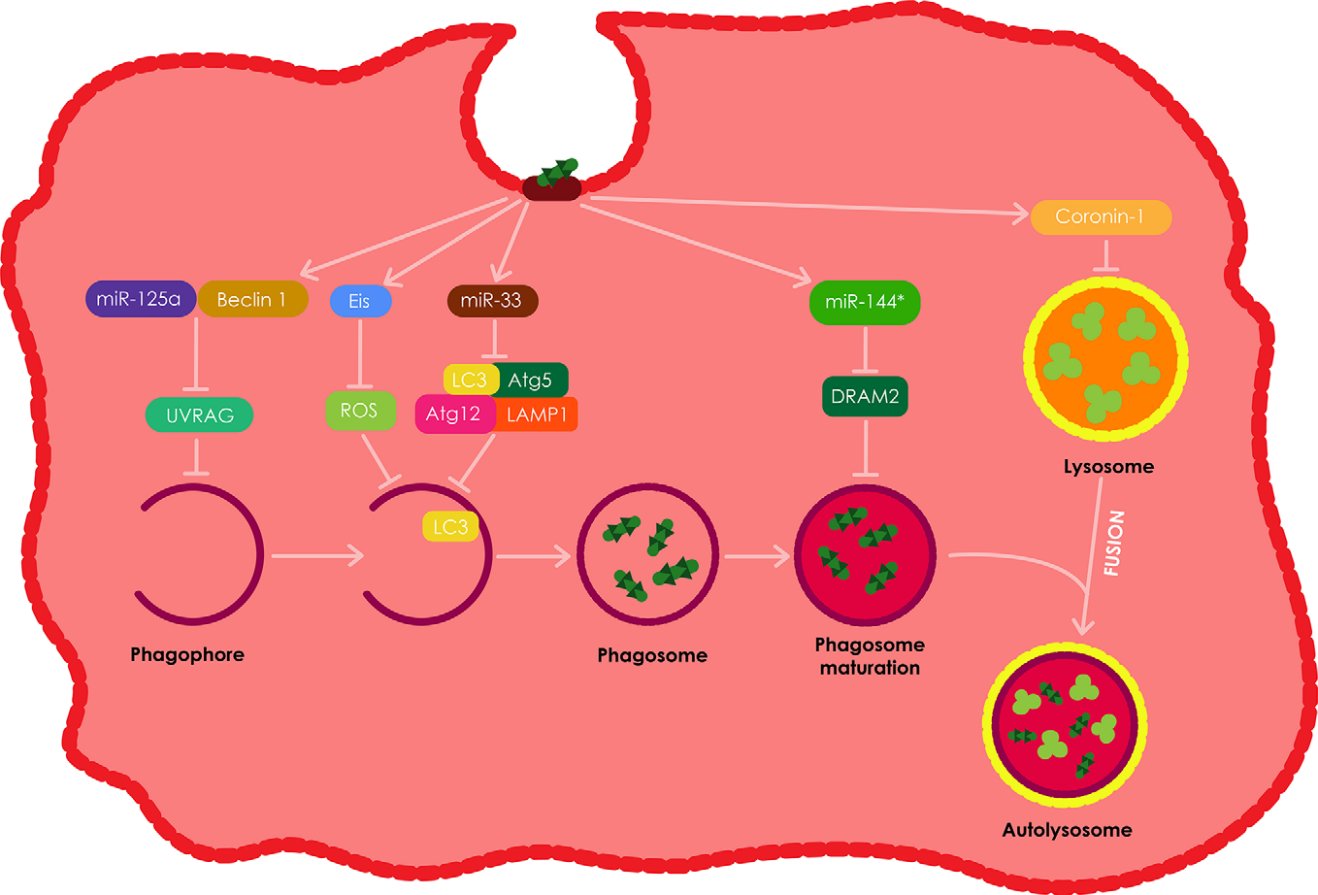
The evasion of autophagy by intracellular *Mtb*. Intracellular *Mtb* have been shown to employ various strategies to evade autophagy in macrophages. Among these, *Mtb* have been shown to induce the production of Eis, which results in the inhibition of ROS and ultimately autophagy. *Mtb* also impedes autophagy by inducing miR-33 expression which in turn supresses the expression of numerous autophagy proteins including; Atg5, Atg12, LC3, and LAMP1. *Mtb* also induces the expression of miR-125a which forms a complex with Beclin-1 resulting in the inhibition of UVRAG and ultimately the inhibition of autophagy in macrophages. *Mtb* also stimulates the production ofmiR-144*, which inhibits DRAM2 resulting in the inhibition of autophagosome maturation. Lastly, intracellular *Mtb* also induces the secretion of coronin1 by theinfected macrophages, which inhibits the fusion of phagosomes with lysosomes. All these autophagy evasion tactics therefore allow for the intracellular survival of *Mtb* in the infected phagocytes.

Intracellular *Mtb* have developed a variety of strategies to overcome the antibacterial defense of the macrophage, including autophagy (Bah and Vergne, 2017). A majority of the uncovered strategies presented in **Figure 7**, are mainly directed at and specifically target distinct steps of xenophagy. *Mtb* have been shown to target the JNK-dependent autophagy by secreting an *N*-acetyltransferase, Eis. However, this type of autophagy does not show sufficient influence on the intracellular growth of the pathogen (Shin et al., 2010; Kim et al., 2012). In addition, both *in vitro* and *in vivo* studies have demonstrated that *Mtb* inhibit the recruitment of NADPH oxidase onto phagosomes that contain the pathogen, thus favoring intracellular growth of *Mtb* (Koster et al., 2017).

### Regulation of miRNA (miR) Targeting Autophagy Machinery

Autophagy is commonly modulated *via* post-translational modification. However, the process can also be modulated at a post-transcriptional level through the expression of numerous small non-coding RNAs (miR). *Mtb* impedes autophagy by down regulating the expression of several autophagy proteins such as Atg5, Atg12, LC3, and LAMP1 by means of inducing miR-33 expression (Ouimet et al., 2016). However, mice with hematopoietic miR-33 deficiency have been demonstrated to retain their ability to control *Mtb* infection. *Mtb* also targets UVRAG through the expression of miR-125a in complex with Beclin-1 resulting in the inhibition of autophagy in macrophages, therefore allowing for the intracellular survival of *Mtb* in the process (Kim et al., 2017).

However, other studies have demonstrated that miR-33 and miR-125a can be induced using PRR ligands separate from *Mtb* (Ouimet et al., 2016; Kim et al., 2017). These findings suggest that these miRs may be expressed as a normal negative feedback loop in host cells to avoid excessive autophagy, which *Mtb* takes advantage of. In addition, *Mtb* stimulates the up-regulation of miR-144* which it uses to decrease the expression of DRAM2, a newly discovered autophagy protein, responsible for promoting autophagosome maturation by activating a second Beclin-1 complex (Kim et al., 2017). Lastly, *Mtb* also promotes the expression of Mc1, which targets and inhibits Bectin-1 complex, by down-regulating the expression of miR-17, thus inhibiting autophagy (Kumar et al., 2016).

### Suppression of the Maturation of Phagolysosomes

The *Mtb* secretes bacterial proteins ATP1/2 (secretion ATPase1/2, secreted secA1/2 protein) and early secretory antigen-6/culture filtrate protein to inhibit the accumulation of vacuolar ATP and GTP enzymes, therefore decreasing intracellular pH, which results in the suppression of phagocyte maturation (Rohde et al., 2007; Zhai et al., 2019). *Mtb* also supresses the formation of lysosomes by inducing an increase in the expression of the coronin 1 protein on the host phagocyte membrane; the extent of the recruitment process and the amount of coronin 1 are proportional to the amount of activated *Mtb* in the microsomes (Schuller et al., 2001; Flynn and Chan, 2003; Zhai et al., 2019).

The IFN cytokine interferon has also been shown to suppress phagocyte maturation through the induction of IL-10 production in a STAT1-dependent manner. IL-10 inhibits the caspase1-dependent IL-1 maturation of pleural fluid mononuclear cells by reducing excess IL-1 (Ma et al., 2014). Serine/threonine-protein kinase G (PKnG) has been shown to augment the virulence, metabolism, growth rate, and drug resistance of *Mtb* by increasing the infectivity of *Mtb* lowering the expression of GlpK and ALD, increasing the expression of Ag85A and Ag85C, and supressing the maturation of lysosomes. PKnG also hinder the fusion of phagosomes and lysosomes by increasing signal transduction in host cells (Walburger et al., 2004; Wong et al., 2013).

### Obstruction of Autophagosome–Lysosome Fusion

As described earlier*, Mtb* can inhibit autophagosome maturation using miR-144*, although, the bacterial factors involved in that process are still unidentified (Kumar et al., 2016). A number of studies have demonstrated that a vital *Mtb* virulence factor called Esat-6 is involved in the process of inhibiting autophagosome maturation in both macrophages and dendritic cells (Romagnoli et al., 2012; Chandra et al., 2015). The exact role of Esat-6 in the process is still unknown but it is estimated that it might be partially facilitated by miR modulation (Kumar et al., 2015). A number of other bacterial pathogens such as *Chlamydia trachomatis*, *Yersinia pestis*, and *Y. Pseudotuberculosis* can also block autophagosome maturation to insure their intracellular survival or growth in macrophages, and similar to *Mtb* their molecular mechanisms involved in the process remain unknown (Pujol et al., 2009; Moreau et al., 2010; Yasir et al., 2011). Mannosylated beads hinder phagosome maturation post phagocytosis, indicating the plausible influence of glycolipids on the survival of intracellular mycobacterium (Astarie-Dequeker et al., 1999; Kang et al., 2005).

The pro-inflammatory transcription factor NF-kB has been shown to control the release of lysosomal enzymes into phagosomes, therefore regulating the killing of intracellular pathogens. In addition, the fusion of phagolysosomes during infection is also regulated by NF-kB, by increasing the production of membrane transport molecules (Gutierrez et al., 2008). The fusion between phagosomes and lysosomes is also suppressed through the calmodulin dependent production of phosphatidylinositol-3-phosphate (PI3P), lower biosynthesis and *via* trafficking the toxin lipoarabinomannan (LAM) after *Mtb* infection (Vergne et al., 2003). As mentioned above, *Mtb* can survive and persist in macrophages by obstracting the fusion between the lysosome and phagosome (**Figure 7**). In DCs, DC-specific intercellular adhesion molecule-3 grabbing nonintegrin (DC-SIGN) facilitates the entry of *Mtb* by interacting with the mycobacterial cell wall component ManLAM. The interaction between DC-SIGN and ManLAM leads to the supression of DC maturation (Geijtenbeek et al., 2003; Tailleux et al., 2003).

## Supression of the Acidification of Phagolysosomes

*Mtb* inhibits phagosome acidification and persists by maintaining a relatively lower acidic intracellular environment (pH~6.2). *Mtb* initiates this process by changing the composition of the phagosome; as the structure and particular molecules on the cell wall function as a barrier that allows macrophages to retain a neutral pH (Chen et al., 2015). This step is followed by encoding the protein tyrosine kinase A (PtkA) in the same operon as PtpA. IFN-γ inducible nitric oxide synthase 2 has been shown to directly activate *Mtb* infected macrophages, resulting in the suppression of the replication of intracellular *Mtb*. LRG-47, a member of the 47-kD guanosine triphosphate familyhas also been demonstrated to play a protective nitric oxide synthase 2 independent role in macrophages against disease. As a result, LRG-47 defient macrophages have a reduced response to *Mtb* due to an inability to completely acidify (MacMicking et al., 2003).

## Inhibition of the Function of Oxidative Species

Oxidative stress refers to a state of imbalance between the pro and antioxidants. Together with the inhibition of macrophage phagocytosis, lysosome maturation and acidification, inhibition of oxidative stress is mandatory process toward archieving *Mtb* latency in the host cells. Oxidative stress can induce lipid peroxidation, protein oxidation, and cause damage to DNA bases. The mitochondria and the NADPH oxidase (NOX) are the two main producers of ROS required to induce autophagy (Shin et al., 2010). NOX plays a key role in the elimination of pathogens and the NOX deficiency in mice has been shown to impair the mice’s ability to eradicate a number of pathogens (Fang, 2004).

However, *Mtb* has been shown to have the ability to deactivate NOX2-derived ROS (Miller et al., 2010), although it is still unclear if there is increased susceptibility to TB in patients with chronic granulomatous disease (CGD) that causes ROS defects as a result of genetic defects in NADPH oxidase. This is because patients with CGD are almost only identified and diagnosed in developed countries where there is a very low prevalence of TB, therefore it is unknown if these patients have increased susceptibility or show a flawed autophagy process against *Mtb* in countries with a high prevalence of TB (Songane et al., 2012). The presence of SigH, an *Mtb* alternative sigma factore induced by heat, oxidative and nitric oxide stresses, has been shown to becompulsory for long lasting *Mtb* infection. The interaction of host innate immune responses with *Mtb* is also regulated by a SigH-dependent regulon (Dutta et al., 2012).

Mycothiol (MSH), a major thiol in *Mtb*, has been shown to have antioxidant activity and helps *Mtb* persist in macrophages through the detoxification of various intracellular compounds. The gene mshD encodes for mycothiol synthase, the final enzyme in the biosynthesis of MSH. A strain of *Mtb* devoid of an mshD gene has been shown to be sensitive to H_2_O_2_ and also displays poor growth on catalase and oleic acid deficient agar plates (Buchmeier et al., 2006). Polymorphonuclear neutrophils (PMNs) eliminate pathogens and are key components in the first line of defense against *Mtb* through the induction of ROS production (Schuller et al., 2001). The enzymes KatG and TrxB2 have been shown to help *Mtb* with stand the oxidative environment and the expression of their related genes are notably increased by H_2_O_2_ and NO (Lamichhane, 2011).

In addition, *Mtb* also produces the protein Eis to enhance its intracellular survival through the blockade of ROS production, thus inhibiting macrophage inflammatory responses, cell death and autophagy (Shin et al., 2010). The Eis protein has been located in the cytoplasm and in *Mtb* containing phagosomes of infected macrophages (Samuel et al., 2007). In addition, traces of anti-Eis antibodies have also been detected in sera obtained from patients with pulmonary TB, thus signifying that Eis is produced during *Mtb* infection in humans (Dahl et al., 2001). Finally, Eis deficient *Mtb* infections have been shown to induce increased caspase-dependent cell death in the host cells, however, this cell death can be inhibited by blocking autophagy and c-Jun N-terminal kinase ROS signaling (Shin et al., 2010).

## Autophagy Inducing Compounds in *Mtb* Infection

The stimulation of autophagy using carbamazepine, rapamycin, small molecule enhancers of rapamycin (SMERs), specifically SMERs 18 and 28, as well as vitamin D_3_ has been shown to induce *Mtb* killing in macrophages. In addition, apart from reducing bacterial burden, carbamazepine has also been shown to stimulate adaptive immunity and improve lung pathology in mice with MDR-TB (Floto et al., 2007; Schiebler et al., 2015; Paik et al., 2019). Using both chemical and RNAi screening, nortriptyline was revealed to be one of the majorcompounds that modulate intracellular mycobacteria and was shown to considerably reduce the survival of intracellular *Mtb* in macrophages through the induction of autophagy (Sundaramurthy et al., 2013). Nitazoxanide (antiprotozoan drug) and flubendazole (antihelminthic drug) were revealed to activate autophagy to limit both bacterial and HIV infections in macrophages (Lam et al., 2012; Chauhan et al., 2015a).

Trehalose has also been reported to induce bacterial killing by activating autophagy and xenophagy in *Mtb* infected macrophages (Sharma et al., 2017). In addition, trehalose has also been shown to be effective againstan HIV and *Mtb* co-infection and regulates the survival of *Mtb* by overriding the HIV mediated blockade of autophagy (Sharma et al., 2017). Vitamin D_3_ was also shown to induce autophagy to facilitate the killing of *Mtb* amidst an HIV co-infection (Campbell and Spector, 2012). Various host factors have been studied as potential anti-*Mtb* therapeutics and have been reported to regulate autophagy and xenophagy in host cells. For example, the compound saracatinib (AZD0530) was revealed to induce autophagy and lysosomal maturation to facilitate the killing of *Mtb* through inhibiting the Src kinase in host cells (Chandra et al., 2016a; Chandra et al., 2016b).

Fluoxetine, a selective serotonin re-uptake inhibitor, has been shown to induce autophagy to restrict the survival of intracellular *Mtb*, possibly by increasing the secretion of TNF-α in *Mtb* infected macrophages. Baicalin also induces anti-inflammatory and antimicrobial activity against *Mtb* infection, by activating autophagy through the PI3K/Akt/mTOR pathway in host cells (Paik et al., 2019). Antimycobacterial agents; loperamide and verapamil, have been revealed to induce autophagy, to facilitate the inhibition of intracellular *Mtb* survival in murine alveolar macrophages. Ambroxol has also been shown to induce autophagy *in vitro* and *in vivo*, resulting in the killing of *Mtb* in murine macrophages (Paik et al., 2019) **Table 1**.

**Table 1:** List of some known authophagy inducing compounds and their applications in TB treatment.

AICs	Mechanism of action	References
Carbamazepine	Neutralization of TNF-α activity	(Schiebler et al., 2015)
Vitamin D_3_	Upregulation of the expression of Atg5 and Beclin-1. Plus, stimulation of the production of CAMP, DEFB4, reactive oxygen and nitrogen intermediates.	(Yuk et al., 2009; Campbell and Spector, 2012)
SMERs 18 and 28	Induction of autophagosome formation by activating Atg5.	(Floto et al., 2007)
Rapamycin	Inhibition of the activity TOR proteins (e.g. mTOR in mammals).	(Floto et al., 2007)
Baicalin	Activation of the PI3K/Akt/mTOR pathway and inhibition of the PI3K/Akt/NF-κB signal pathway.	(Zhang et al., 2017)
AZD0530	Inhibition of Src kinase activity.	(Chandra et al., 2016a; Chandra et al., 2016b)
Fluoxetine	Increase the production of TNF-α.	(Stanley et al., 2014)
Ambroxol	Activate of Atg5 (possibly).	(Choi et al., 2018)
loperamide	Reduction of TNF-α production and p62 degradation.	(Juárez et al., 2016)
Trehalose	Activation of PIKFYVE resulting in TFEB nuclear translocation in a MCOLN1-dependent manner.	(Sharma et al., 2020)

In addition, the interaction of zymosan with macrophage TLRs or the Fc_γ_Rs during phagocytosis has been shown to promote the recruitment of the LC3 autophagy protein to phagosomes. Both TLRs and Fc_γ_Rs activate NOX2 NADPH oxidase, which is known to induce the production of reactive oxygen species (ROS). NOX2-dependent ROS production has been shown to either induce or aid in LC3 recruitment to phagosomes, thus promoting phagosome maturation and ultimately microbial killing (Huang et al., 2009; Yang et al., 2012).

## Nanotechnology Approaches in HDT-TB

**Figure 8:**
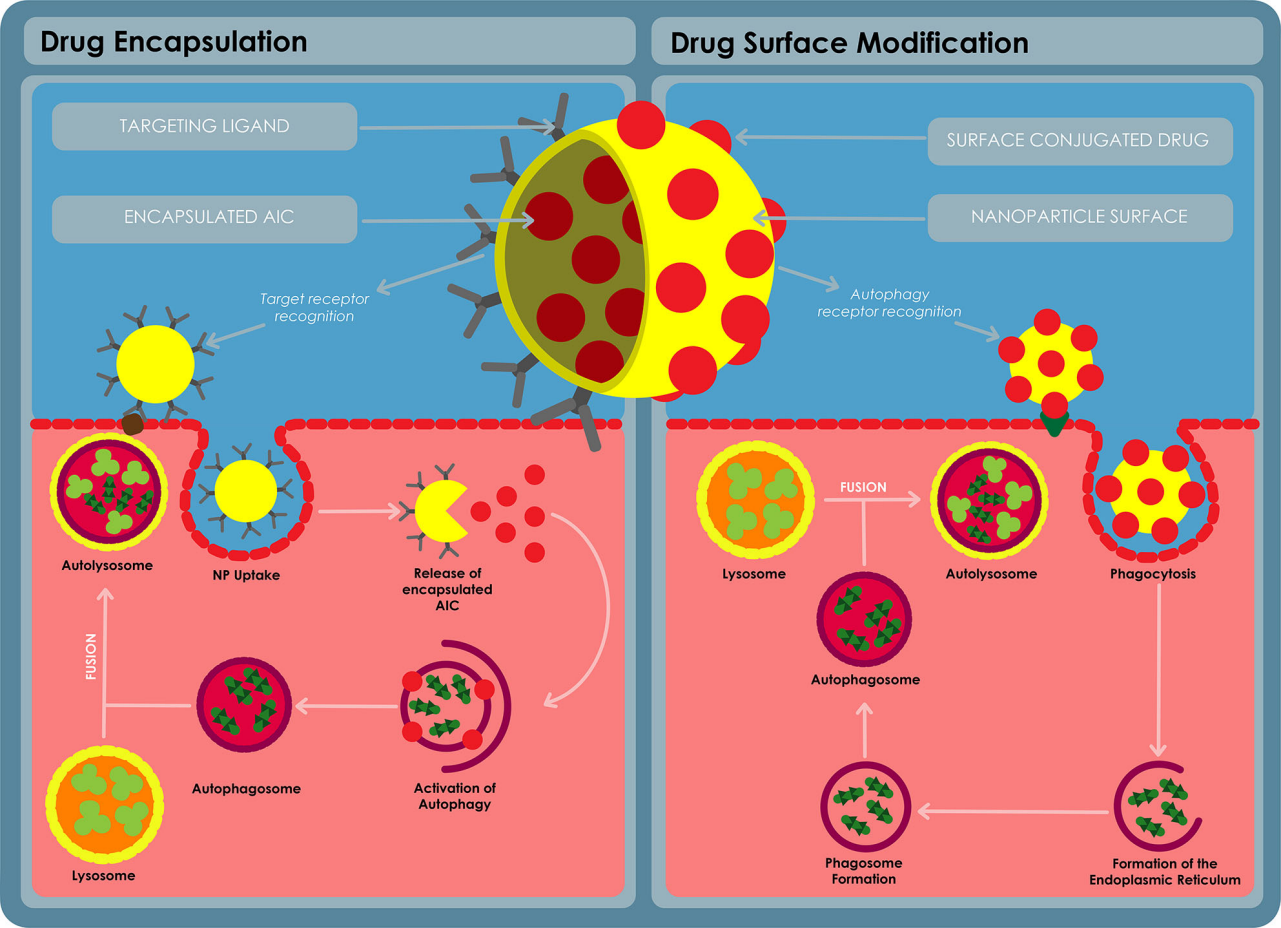
A diagram for autophagy inducing nanomedicine. The nanomedicine can be formulated in a variety of ways, the AIC can be encapsulated on the inside of a nanoparticle and released at the target site using a ligand to target the delivery nanosystem into the target cells. The AIC can also be conjugated on the surface of the nanoparticle and used to target the AIC to the target cells using receptor recognition. The AIC can stimulate autophagy *via* receptor stimulation or the AIC can stimulate specific autophagy pathways after being taken up by the cells.

In this decade, NPs have received significant attention in disease therapy (Mallakpour, 2016). NPs are defined as engineered particles with a size range typically of 1–100 nm and also up to 1000 nm (Nagavarma et al., 2012). NPs can be classified into various categories as either organic (e.g. liposomes, polymeric NPs, lipid NPs or dendrimers), metallic (iron oxide Nps, gold NPs (GNPs), silver NPs, quantum dots) or ceramic (silica NPs, calcium carbonate NPs). The application of NPs toward drug delivery and diagnostics is known as nanomedicine. As drug delivery systems, drugs can either be encapsulated within the NP or covalently attached onto the surface of the NP (**Figure 8**). By so doing, the properties of these drugs can be improved, e.g. the aqeous solubility, chemical stability and translocation across biological barriers. The release of the drugs can also be controlled to occur over a desired time course or to occur at target sites following an external trigger (e.g. pH orenzymatic action) thus modulating plasma and intracellular pharmacokinetics of the drugs (Dube et al., 2013). Cells also take up NPs more efficiently making NPs a more auspicious transport and delivery system (Nasiruddin et al., 2017).

NPs have been observed to induce ROS production in various cell types (Yu et al., 2014; Chiu et al., 2015; Guo et al., 2015). Spherical silica NPs of 60 nm in size were shown to stimulate ROS generation in (HepG2) cells at a final concentration of 100 μg/ml (Yu et al., 2014). Amine-modified but not pristine or carboxylate modified 60 nm polystyrene spheres were also observed to induce the generation of ROS in both BEAS-2B lung epithelial cells and RAW264.7 macrophages atconcentrations of 20 μg/ml. The amine-modified spheres also induced an increase of LC3 in both of the cells lines (Chiu et al., 2015). RAW264.7 macrophages were observed to use phagocytic pathways to take up elongated pristine iron oxide nanorods (1000 nm × 100 nm), which are then distributed in the cytosol by autophagosome-like vacuoles 24h post exposure to cells (Park et al., 2014). Other studies have revealed that these nanorods also induce an increase in ROS and nitrous oxidelevels in RAW264.7 macrophages. In addition, the nanorods also induced an increase in the expression levels of Atg5, Beclin1, Erk, p62, and LC3-II, while reducing JNK phosphorylation levels in the same cell line (Xia et al., 1995; Nordstrom et al., 2009).

GNPs of size 5 and 13 nm were observed to enhance ROS production in hypoxic HK-2 cells and increase autophagy in hypoxic cells. To study the relationship between apoptosis and autophagy, 3-methyladenine (3-MA), a well-known autophagy inhibitor was used. 3-MA significantly inhibited the GNP induced autophagy as expected. However, this treatment resulted in a 36% reduction in the viability and a 29% enhancement in the apoptosis of GNP treated cells under normoxic conditions. On the other hand, exposure to GNPs resulted in 61% increase in viability and a 52% decrease in the apoptosis of in HK-2 cells under hypoxic conditions (Ding et al., 2014).

Silica NPs at 500 μg/ml, were observed to induce the highest TNF-α release RAW264.7 macrophages, followed by poly(lactic-co-glycolic acid) (PLGA) NPs which induced the release of significantly lower TNF-α and finally, silk NPswhich induced the lowest release of TNF-α in RAW264.7 macrophages (Saborano et al., 2017). Unloaded Eudragit^®^ RS NPs have been observed to be cytotoxic to NR8383 rat macrophages exposed to doses varying from 15 - 100 µg/mL, the internalized NPs were also shown to reach and alter the structure of the mitochondria. In addition, exposure to the NPs also triggered an increase in ROS production and autophagy in the macrophages (Eidi et al., 2012).

Particulate β-glucans (WGP) derived from the yeast *Sacchromyces cerevisiae* have also been shown to induce LC3 recruitment by increasing LC3-II expression. Formation of an autophagosome in WGP-induced DCs was also detected, indicating that the binding of WGP to dectin-1 in DCs, triggers both autophagy and LC3-associated phagocytosis. In addition, autophagy induced TNF-α production in WGP treated DCs, but had no influence on the secretion of IL-12, suggesting that the maturation of autophagy induced DCs may be archived using a unique pathway. Furthermore, the WGP also reduced iNOS expression in autophagy deficient DCs. WGP treated DCs also induced greater proliferation in CD4 ^+^T-cells when compared to autophagy deficient DCs (Ding et al., 2019).

In a study demonstrating autophagy in *Mtb* infected macrophages (HDT-TB), Upadhyay et al. (2019) reported the capacity of yeast derived glucan particles (GP) loaded with high payload of rifabutin (RB) NPs [(RB-NPs)-GP] to induce anti-mycobacterial and cellular activation responses in *Mtb* infected J774 macrophage cells. The exposure to (RB-NPs)-GP was observed to trigger strong innate immune responses in *Mtb* infected macrophages including the induction of apoptosis, autophagy as well as reactive oxygen and nitrogen species. In addition, the (RB-NPs)-GP formulation was found to induce a 2.5 fold increase in the efficacy of the RB drug (Upadhyay et al., 2019). However, additional investigations *in vivo* as well as studies to target and deliver known AICs are required.

## Conclusion and Future Directions

The interaction between *Mtb* and the host immunity has been shown to determine the outcome of the infection. The innate defense mechanisms of the host play a pivotal role in the process. An overlap was observed between the pathways that induce apoptosis and autophagy, for example *Mtb* has been shown to stimulate the production of TNF in *Mtb* infected macrophages through the TLR2-mediated signaling pathway. In turn, the induction of TNF-α activity leads to apoptosis in *Mtb* infected cell. On the other hand, the production of TNF-α has also been shown to be vital in the instigation of autophagy against *Mtb* infection. In addition, TRL2 has also been shown to induce mitochondrial ROS in infected macrophages resulting in both apoptosis and/or autophagy.

Nonetheless, *Mtb* has developed various mechanisms to evade the host’s protective immunity and the increase of *Mtb’s* resistance to a variety of existing antibiotics has become a threat to public health across the world. Therefore, the requirement for new therapeutic strategies has become a key priority. A promising approach is the use of AICs as part of HDT. This strategy can be coupled with nanotechnology, to improve the delivery of these compounds or to synthesize novel nanosized HDT-TB therapeutics, as studies have revealed that various NPs can induce autophagy in a variety of cell types. In addition, the introduction of NPs to HDT-TB can allow for targeted drug delivery, therefore minimizing the toxic side effects, while increasing the amount of the drugs that reach target cells.

However, despite the wide array of available AICs and NPs, there remains a very limited amount of research regarding the application of AICs and therapeutic NPs against *Mtb*. Mechanistic aspects of the macrophage response to *Mtb* and its suppression of autophagy, could be exploited to rationally design nanomedicines against this infection. The efficacy of nanomedicines to eradicate intracellular *Mtb* within the granuloma structureis also yet to be investigated. Macrophages have typically been the target in TB immunotherapy, however, DCs should also be targeted, as they are the bridge between the innate and adaptive immunity and can therefore be targeted for the purpose of both vaccination and therapy. The use of animal models is also required to determine efficacy of NP HDT in pre-clinical studies. Toxicity of the selected NPs is also a key concern and thus, we advocate for the use of biodegradable NPs as they have been shown to have minimum toxicity *in vitro.* Ultimately, we predict that greater understanding of both TB pathogenesis and the mode of action of AICs will reveal greater insights toward designing a superior treatment strategy against *Mtb* infections in the future.

## Author Contributions

RM wrote the full review paper. MM and AD co-supervised, guided, and helped shape the paper and also edited the paper before the paper was submitted. All authors contributed to the article and approved the submitted version.

## Funding

Research reported in this publication was supported by the Fogarty International Center of the National Institutes of Health under Award Number K43TW010371 granted to AD and by National Research Foundation (NRF) of South Africa, award number UID 111887 awarded to RM. The content is solely the responsibility of the authors and does not necessarily represent the official views of the National Institutes of Health.

## Conflict of Interest

The authors declare that the research was conducted in the absence of any commercial or financial relationships that could be construed as a potential conflict of interest.
